# The use of CytoSorb® in a prasugrel‐loaded patient undergoing emergent coronary artery bypass graft surgery

**DOI:** 10.1002/ccr3.6990

**Published:** 2023-03-19

**Authors:** Erich Moresco, Hannes Dejaco, Volker Humbert, Matthias Thoma

**Affiliations:** ^1^ Department of Anesthesiology and Intensive Care Medicine Medical University of Innsbruck Innsbruck Austria; ^2^ CytoSorbents Europe GmbH Berlin Germany

**Keywords:** coronary artery bypass graft surgery, CytoSorb®, perioperative bleeding, prasugrel, multiplate, platelet inhibition

## Abstract

Patients with acute coronary syndrome (ACS), failed percutaneous coronary intervention (PCI) and with an indication for emergent coronary artery bypass graft surgery (CABG) are at an increased risk of perioperative bleeding. Integration of the CytoSorb® (CS) adsorber into the cardiopulmonary bypass appears to bind prasugrel and minimize the risk of perioperative bleeding.

## Abstract 2

Intraoperative use of CytoSorb hemoadsorption therapy in prasugrel‐loaded patients undergoing emergency cardiac surgery might be an effective option to reduce the risk of bleeding complications. Further investigations, however, are needed for a better understanding of the precise role of the adsorber in this setting.

## 1 INTRODUCTION

Prasugrel is a thienopyridine‐type antiplatelet prodrug, which is converted into its active metabolite R‐138727 after rapid and nearly complete (>80%) absorption from the gut.^1^ The maximum plasma concentration is achieved within 30 min.^2^ The molecules are highly bound to human albumin, with only a fraction (2%) remaining unbound and therefore active. The active metabolite of prasugrel binds irreversibly to the platelets, thus inhibiting their function. It is administered in combination with acetylsalicylic acid (ASA) to patients who suffer from acute coronary syndrome (ACS) and are scheduled for percutaneous coronary intervention (PCI). The average plasma half‐life of the active metabolite is about 4 h, and it is primarily excreted in the urine and in a small part in the feces. It seems that prasugrel metabolism does not vary significantly among individuals.^3^ CytoSorb® (CS; CytoSorbents Corporation) is an adsorption device used for removing inflammatory toxins from the body. In addition to other indications (e.g. rhabdomyolysis^4^), CS is also used in antithrombotic drug removal with increasing frequency. In this case report, we show the potential benefit of CS for reducing bleeding complications associated with cardiopulmonary bypass in a patient who was preloaded with prasugrel and ASA.

## 2 CASE REPORT

We describe the case of a 63‐year‐old male patient with a positive history of arterial hypertension suffering from acute chest pain. He admitted himself to the accident and emergency department (A&E) where an Electrocardiogram (ECG) and blood tests were done. Prior to the admission, the patient took 200 mg of ASA. ECG showed signs of an acute ST‐elevation myocardial infarction (STEMI) and blood results were also pathological (troponin T 4607 ng/L [range 0–14 ng/L] creatine kinase [CK] 2895 U/L [range 38–190 U/L]). Based on the clinical and laboratory findings, emergency coronary angiography was performed. Before undergoing cardiac catheterization, the patient was administered 250 mg acetylsalicylic acid (ASA), 5000 units of unfractionated heparin intravenously (i.v.), and 60 mg of prasugrel per os. Coronary angiography showed severe multi‐vessel coronary artery disease with 99% stenosis of the left anterior descending artery (LAD), 90% stenosis of the ramus diagonalis, 99% stenosis of the ramus intermedius, and 70% stenosis of the circumflex artery shortly before branching into the ramus marginalis as the major findings. At the discretion of the interventional cardiologist and the cardiac surgeon on call, the clinical decision was made to abort the percutaneous intervention and to convert to emergency open‐heart surgery, which was started a few hours later.

To reduce the risk of bleeding during and especially after open‐heart surgery given the previous loading with prasugrel, a CS adsorber was integrated into the cardiopulmonary bypass (CPB, S5 Heart‐Lung Machine; LivaNova PLC). Platelet function was monitored using Multiplate® analyzer (Roche Diagnostics) before, during, and on the first postoperative day (see Table 1).

**TABLE 1:** Multiplate® values measured before, after CBP, and postoperative.

Multiplate®	Before CPB (U)	After CPB (U)	First postoperative control (U)	Second postoperative control (U)	First postoperative day (U)
ASA monitoring	33	16	11	10	19
P2Y12 antagonist monitoring	16	15	9	13	16
TRAP	74	41	39	77	40

*Note*: ASA reference range/therapeutic range: no aspirin medication: 72–138 U; Aspirin therapy: 0–30 U; P2Y12 reference range/therapeutic range: No P2Y12 antagonist medication: 48–113 U; P2Y12 antagonist medication: 0–47 U. TRAP: reference range without antiplatelet medication: 82–161 U; Reference range under aspirin administration: 69–150 U; Reference range under P2Y12 antagonist administration: 57–137 U; Reference range with aspirin and P2Y antagonist administration: 42–144 U.

Abbreviations: ASA, acetylsalicylic acid; CBP, cardiopulmonary bypass; TRAP, thrombin receptor activating peptide.

CPB and ACC (aortic cross‐clamp) times were 112 and 81 minutes respectively. The following four grafts were installed: Left Internal Mammary Artery (LIMA) to LAD, saphenous vein graft to ramus diagonalis, saphenous vein graft to ramus intermedius, and saphenous vein graft to ramus marginalis. After CPB was completed, two platelet concentrates, each 300 mL, were transfused as well as approximately 370 mL of intraoperative blood salvage. Postoperatively the patient was transferred to the intensive care unit (ICU) for further monitoring and treatment.

During his postoperative stay in the ICU, only minor blood loss was recorded, although platelet function still seemed to be inhibited according to the Multiplate® results (see Table 1). Due to iatrogenic hemodilution, the patient received 1 unit of packed red blood cells to keep his hemoglobin level above 8 g/dL, as well as 4 g fibrinogen concentrate due to reduced FIBTEM ® values measured with rotational thromboelastometry (ROTEM®). On the second postoperative day, administration of ASA was recommenced after any contraindications had been ruled out. Low molecular heparin was also given according to our hospital standard protocol to prevent thrombosis. On the same day, the patient could be transferred to the intermediate care unit (IMCU) of the cardiac surgery department. The discharge from hospital was on the sixth day after surgery.

## 3 DISCUSSION

To date (October 2022), there have been no publications on the use of CS in the setting of prasugrel‐loaded patients undergoing emergent cardiac surgery. However, the use of CS for removing ticagrelor, a reversible binding antiplatelet drug, and rivaroxaban in patients undergoing CABG has already been described with beneficial effects regarding perioperative and postoperative outcomes.^5^, ^6^, ^7^

In our case, CS was only used during cardiopulmonary bypass and it remains speculative to which extent the relevant metabolites of Prasugrel were adsorbed by the device. The irreversible nature of the platelet blockade explains why even complete removal of the antithrombotic does not result in complete restoration of platelet function and so platelet administration is likely to be necessary, as was the case in our patient. Maybe this aspect also explains the Multiplate results ® that showed some inhibition up to the second postoperative day, or there was some impact of CPB on these results, and the fact that there is generally a poor correlation of post‐CPB MEA results and bleeding as discussed in the literature.^8^ Effects of CytoSorb on ASA are not to be expected, however, the impact of ASA on bleeding complications might also be quite small in comparison to the effect of Prasugrel platelet inhibition. In summary, our patient did not experience significant intra‐ or postoperative bleeding, and in our opinion, the postoperative administration of 1 erythrocyte concentrate, as well as the fibrinogen replacement was for prophylactic and not therapeutic reasons. So overall, the postoperative blood loss or the need for transfusions, respectively, were much lower than we had expected in this setting.

Obviously, there are several limitations to this case report. First, we did not measure any plasma levels of the active metabolites of prasugrel and can therefore only speculate on any adsorption of the substance by the adsorber and so on the direct impact of CytoSorb in this case. Second, a single case report does not allow for any general conclusions, and a more structured investigation of this indication is required.

CS has been shown to be associated with a significantly reduced need for blood cell transfusions in the intraoperative setting during urgent infective endocarditis surgery.^9^ So even without the presence of antithrombotic substances, the use of the adsorber might have an effect concerning reduction in bleeding. This might be because of the reduction in inflammatory substances and therefore the reduction of prostaglandin synthesis, but a better understanding of the precise mechanisms behind this is needed.

## 4 CONCLUSION

We assume that intraoperative use of CS might reduce the risk of bleeding also in Prasugrel‐loaded patients undergoing emergent cardiac surgery. However, the gathered data cannot confirm a clear causal relationship to the use of CS, and clinical trials are required to investigate this indication further.

## AUTHOR CONTRIBUTIONS

EM, HD, VH, and MT contributed to conception and design; EM, HD, VH, and MT contributed to the analysis; EM and MT contributed to data acquisition. EM and MT drafted the manuscript. All authors critically revised the manuscript, contributed to interpretation, gave final approval, and agreed to be accountable for all aspects of the work ensuring integrity and accuracy.

## FUNDING INFORMATION

The publication of this work is supported by Cytosorbents Europe GmbH, Berlin, Germany.

## CONFLICT OF INTEREST STATEMENT

The authors declare no potential conflict of interest with respect to the research, authorship, and/or publication of this article.

## ETHICAL APPROVAL

Not applicable.

## CONSENT

Written informed consent was obtained from the patient for publication of this case report. A copy of the written consent is available for review by the Editor in Chief of this journal upon request.

## Data Availability

Data are available upon reasonable request.
